# MicroRNA-29b/142-5p contribute to the pathogenesis of biliary atresia by regulating the *IFN*-γ gene

**DOI:** 10.1038/s41419-018-0605-y

**Published:** 2018-05-10

**Authors:** Yifan Yang, Zhu Jin, Rui Dong, Chao Zheng, Yanlei Huang, Yijie Zheng, Zhen Shen, Gong Chen, Xiaoying Luo, Shan Zheng

**Affiliations:** 1 Department of Pediatric Surgery, Children’s Hospital of Fudan University, and Key Laboratory of Neonatal Disease, Ministry of Health, Shanghai, China; 2 Medical Scientific Liaison Asian Pacific, Abbott Diagnostics Division, Abbott Laboratories, Shanghai, 200032 China; 3 State Key Laboratory of Oncogenes and Related Genes, Shanghai Cancer Institute, Renji Hospital, Shanghai Jiao Tong University School of Medicine, Shanghai, China

## Abstract

Biliary atresia is one of the most common liver disease in infancy. The cause and pathogenesis remain largely unknown. This study aimed to investigate the potential regulatory effect of miR-29b/142-5p on *IFN-γ* gene methylation. miRNAs microarray was performed on four pairs of liver and blood specimens from biliary atresia and choledochal cysts. We found the overexpression of miR-142-5p and mRNA level of DNA methyltransferase (DNMT) 1, and miR-29b and DNMT3a/DNMT3b were significantly negatively correlated in biliary atresia livers. Meanwhile, the methylation of the LINE-1, ALU and SAT2 repetitive sequences and the *IFN-γ* promoter was lower, but the expression of IFN-γ was upregulated. After transfected with DNMTs siRNAs, downregulation of DNMTs exerted a significant hypomethylating effect on the repetitive sequences, which led to upregulation of IFN-γ in Jurkat cells. The direct interactions between miR-29b and DNMT3a/3b, and miR-142-5p and DNMT1 were identified using luciferase reporter assays. By transfecting mimics of miR-29b/142-5p into Jurkat cells, we found overexpression of miR-29b/142-5p markedly suppressed expression of DNMTs. Furthermore, the methylation of repetitive sequences and the *IFN*-γ promoter region were remarkably downregulated, and with elevated IFN-γ expression. After transfecting the miRNA inhibitors, the levels of DNMTs and the methylation of the *IFN*-γ gene promoter region was upregulated, while levels of IFN-γ were markedly suppressed. Our study suggested that miRNA-29b/142-5p overexpression and targeted inhibition of DNMTs expression resulted in decreased overall gene methylation and overexpression of the methylation-sensitive *IFN*-γ gene.

## Introduction

Biliary atresia is one of the most common causes of obstructive jaundice and the most common liver disease in infancy. It is characterized by progressive destructive hepatobiliary inflammation and fibrous obstruction, intrahepatic biliary fibrosis atresia, and eventually cirrhosis^1, 2^. While the etiology remains unclear, this worldwide problem has plagued innumerable pediatric surgeons.

Some studies have shown that biliary atresia is a type of CD4+ helper T-lymphocyte 1 (Th1)-mediated autoimmune diseases^3–6^. IFN-γ plays a crucial role during this process. Activated CD4+ Th1 cells secrete a large amount of IFN-γ, which induces a strong immune response, eventually leading to intrahepatic and extra-hepatic bile duct injury^5, 6^. The extra-hepatic biliary tract of *Ifn-γ*-/- mice remained open after rotavirus infection. However, *Ifn-γ*-/- mice that were infected with virus and recombinant IFN-γ showed symptoms of biliary atresia, similar to the wild-type mice injected with virus^7^. This suggests that IFN-γ plays a key role in the pathogenesis of biliary atresia in mice. The mechanism of IFN-γ upregulation in the pathogenesis of biliary atresia and its biological function has consequently become the focus of the etiology in biliary atresia.

Recent studies have reported that epigenetics play an important role in the development of biliary atresia. Our previous results indicated that the genomic DNA of CD4+ T lymphocytes in the peripheral blood of children with biliary atresia was generally hypomethylated and that the expression of DNA methyltransferase (DNMT) 1 was significantly decreased. The hypomethylation of the IFN-γ promoter region was negatively correlated with IFN-γ overexpression^8^. Due to the large number of CpG islands in its promoter region serving as sites for methylation, the *IFN-γ* gene is a methylation-sensitive gene^9–11^ Studies have shown that 5-aza-2′-deoxycytidine (5-aza-dC) inhibition of methylation could establish biliary atresia, and that bile duct injury is closely related to IFN-γ expression in a zebrafish model^12^. These findings suggested that DNA hypomethylation is involved in the pathogenesis of biliary atresia, potentially through upregulation of IFN-γ. Additionally, our previous research findings indicated that there were many differentially expressed microRNAs (miRNAs) in children with biliary atresia^13^. These included miR-29b and miR-142-5p which might be related to methylation. Therefore, this study will further investigate the regulatory effect of miR-29b/142-5p on *IFN-γ* gene methylation and its possible molecular mechanism.

## Results

### Identification of miR-29b and miR-142-5p overexpression in biliary atresia

**Fig. 1:**
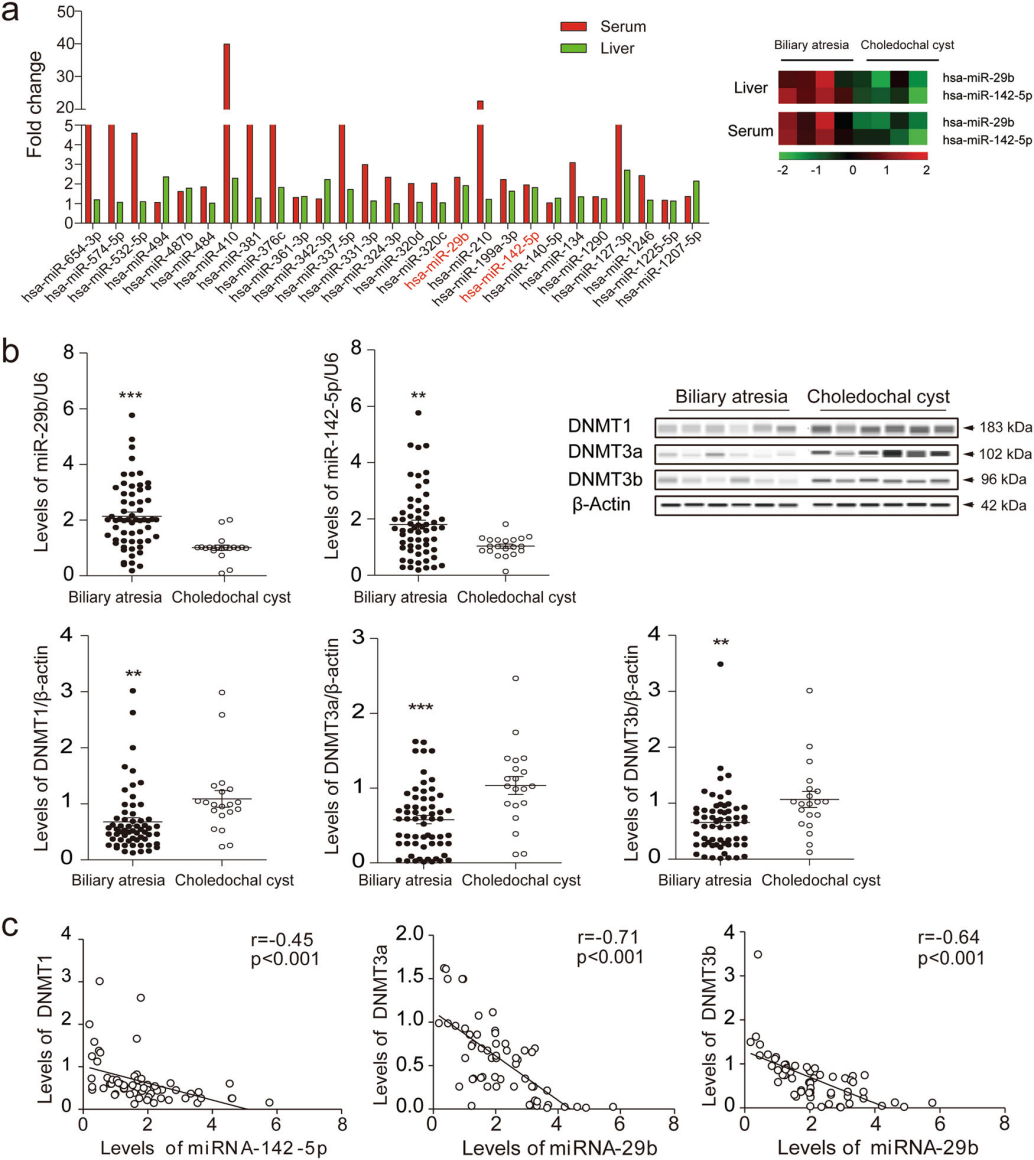
Identification of miR-29b/142-5p overexpression and DNMTs downregulation in biliary atresia. **a** Twenty-seven differentially expressed miRNAs were both upregulated in liver and peripheral blood samples obtained from biliary atresia cases (*p* < 0.05 with fold-change > 1.5) by miRNAs microarray; **b** Levels of miR-29b/142-5p in 60 biliary atresia and 16 choledochal cysts liver samples were detected using qPCR and normalized to U6 levels. Levels of DNMTs were detected by qPCR (normalized to β-actin) and western blotting. **c** The expression levels of miR-142-5p and DNMT1, and miR-29 and DNMT3a/DNMT3b mRNA were significantly negatively correlated (*p* < 0.001, *r* = −0.45, −0.71, −0.64, respectively). **p < 0.01, ***p < 0.001. Date represent mean values ± SD

To identify biliary atresia-specific miRNA profiles, miRNAs microarray detection was performed on four pairs of liver specimens and peripheral blood from biliary atresia and choledochal cysts cases. Fifty-two differentially expressed miRNAs were identified, among which 27 were upregulated (Fig. 1a), 3 were downregulated, and 22 were opposite in expression profiles of liver and peripheral blood samples obtained from biliary atresia cases (*p* < 0.05 with fold-change > 1.5). We further screen the miRNAs that may closely related to DNA methylation from these 30 differentially expressed miRNAs, and target prediction indicated that DNMT 3a and 3b were likely the target genes of miR-29b while DNMT1 was the target gene for miR-142-5p.

For validation, expressions of miR-29b/142-5p and DNMTs in liver samples from 60 biliary atresia and 16 choledochal cysts were examined. We found that the expression of miR-29b and miR-142-5p were remarkably upregulated (*p* < 0.01), and that the messenger RNA (mRNA) and protein levels of DNMTs were downregulated (*p* < 0.05) in biliary atresia cases (Fig. 1b). The expression levels of miR-142-5p and DNMT1, and miR-29 and DNMT3a/DNMT3b mRNA were significantly negatively correlated (*p* < 0.001, Fig. 1c).

### Global genomic hypomethylation induced IFN-γ upregulation in biliary atresia

**Fig. 2:**
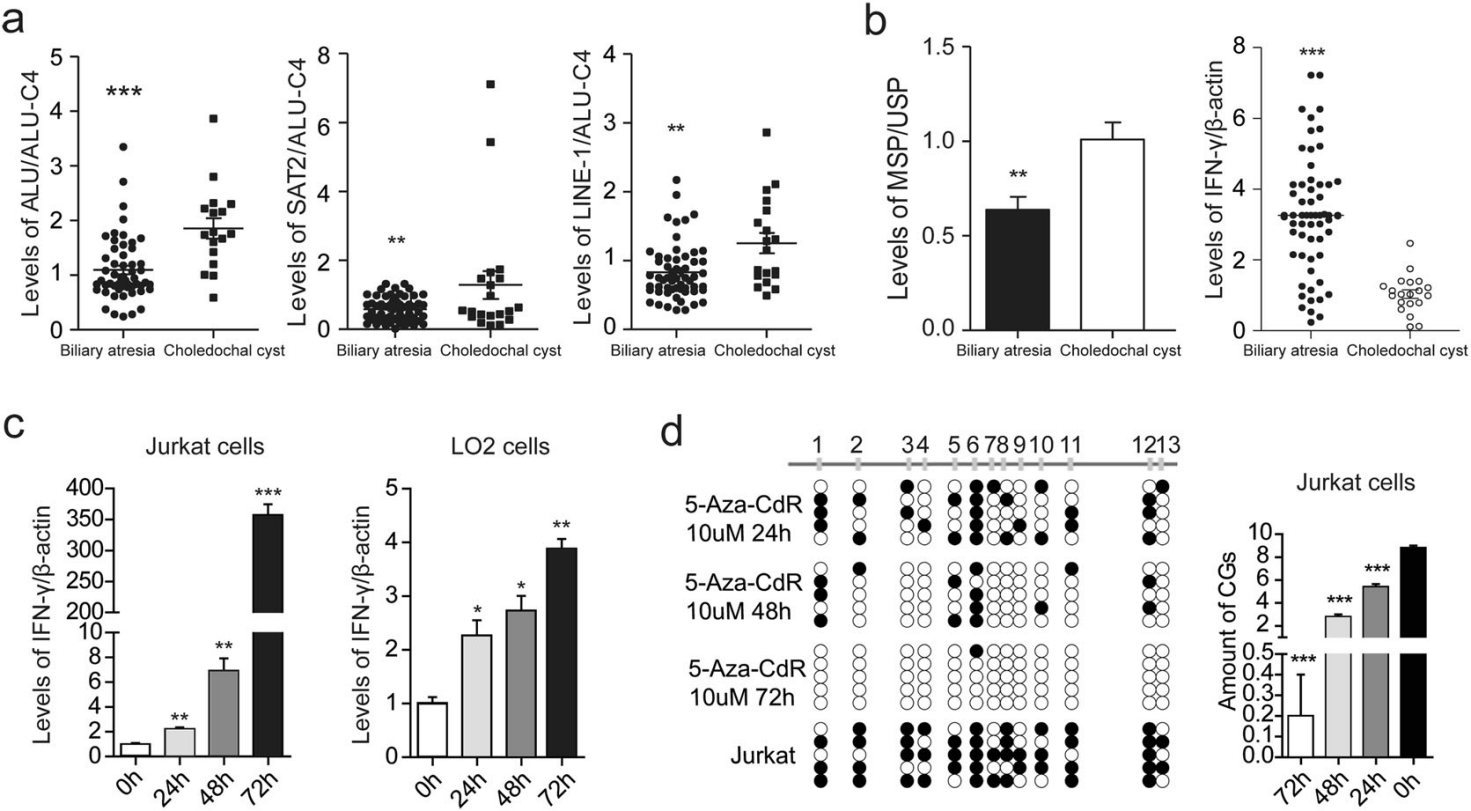
Global genomic hypomethylation induced IFN-γ upregulation in biliary atresia. **a** The quantitative methylation of the LINE-1, ALU, and SAT2 sequences were analyzed by methylation-specific PCR (MS-PCR) and normalized to ALU-C4 in biliary atresia liver samples. **b** The unmethylated (U) or methylated (M) IFN-γ promoter sequence was carried out by MS-PCR in biliary atresia liver samples. Levels of IFN-γ in 60 biliary atresia and 16 choledochal cysts liver samples were detected using qPCR and normalized to β-actin. **c** LO2 cells and Jurkat cells were treated with 5-aza-dC at different time points. Levels of IFN-γ in both cells were detected using qPCR and normalized to β-actin. **d** The methylation of the IFN-γ promoter were detected by bisulfite genomic sequencing at different time points in Jurkat cells. *p < 0.05, **p < 0.01, ***p < 0.001. Date represent mean values ± SD from three independent experiments

The quantitative methylation analysis of the LINE-1, ALU, and SAT2 sequences showed that the methylation levels of these repetitive sequences were lower compared to controls (*p* < 0.01, Fig. 2a). This suggests that genomic DNA was hypomethylated in all biliary atresia livers. Methylation-specific PCR was carried out for the unmethylated (U) or methylated (M) *IFN-γ* promoter sequence. This showed that the *IFN-γ* promoter was hypomethylated (*p* < 0.01, Fig. 2b), and that the expression of IFN-γ was upregulated (*p* < 0.001, Fig. 2b) in biliary atresia cases. To determine the effect of methylation on the *IFN-γ* promoter and the associated expression of IFN-γ, LO2 cells and Jurkat cells were treated with 5-aza-dC at different time points. A significant increase in IFN-γ expression levels were observed with increased times of treatment in both cell lines (*p* < 0.05, Fig. 2c). Notably, an increase of nearly 350-fold was recorded in Jurkat cells (Fig. 2c). We further investigated the methylation of the *IFN-γ* promoter, and found that the ratio of CpG sites decreased with an increased time of treatment in Jurkat cells (*p* < 0.001, Fig. 2d).

### Downregulation of DNMTs contributed to *IFN-γ* promoter hypomethylation

**Fig. 3:**
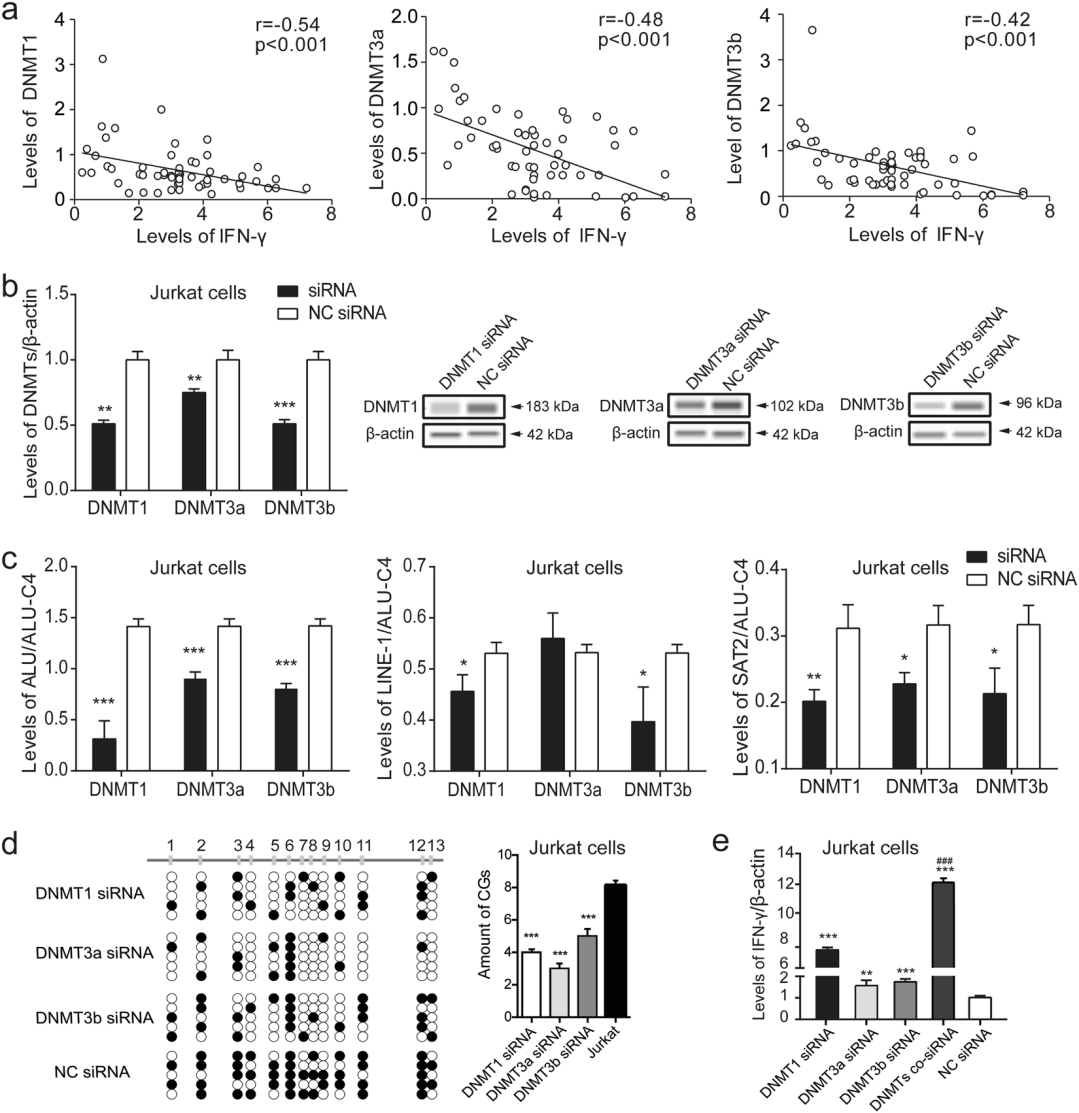
Downregulation of DNMTs contributed to IFN-γ promoter hypomethylation. **a** Levels of DNMTs mRNA were negatively correlated with the mRNA expression of IFN-γ in biliary atresia cases (*p* < 0.001, *r* = −0.54, −0.48, −0.42, respectively). **b** DNMT siRNAs were transfected into Jurkat cells. Levels of DNMTs were detected using qPCR (normalized to β-actin) and western blotting. **c** The quantitative methylation of the LINE-1, ALU, and SAT2 sequences were analyzed and normalized to ALU-C4 in Jurkat cells. **d** The methylation of the IFN-γ promoter were detected by bisulfite genomic sequencing, and **e** levels of IFN-γ were detected using qPCR and normalized to β-actin in Jurkat cells after transfected with DNMT siRNAs. *p < 0.05, **p < 0.01, ***p < 0.001 vs. NC siRNA. ^###^*p* < 0.001 vs. DNMTs siRNA. Date represent mean values ± SD from three independent experiments

In this study, we found that the dramatically decreased mRNA levels of DNMTs were negatively correlated with the mRNA expression of IFN-γ in biliary atresia cases (*p* < 0.001, Fig. 3a). To confirm the suppressive role of DNMTs, DNMTs small-interfering RNA (siRNAs) were transfected into Jurkat cells to silence DNMTs expression. Downregulation of DNMTs exerted a significant hypomethylating effect on the ALU, LINE-1, and SAT2 repetitive sequences (*p* < 0.05, Fig. 3b, c), which induced hypomethylation of the *IFN-γ* promoter and led to upregulation of IFN-γ expression (*p* < 0.01, Fig. 3d, e). Moreover, the levels of IFN-γ mRNA were elevated 12-fold in Jurkat cells transfected with three DNMT siRNAs, comparing the NC siRNA group (Fig. 3e).

### miR-29b/142-5p overexpression led to IFN-γ upregulation by targeting DNMTs

To confirm the target gene of miR-29b/142-5p, luciferase reporter assays were performed. Transfection with mimics of miR-29b and miR-142-5p could respectively inhibit the DNMTs wild-type vectors luciferase activity (*p* < 0.05), but failed to inhibit DNMTs mutant vectors luciferase activity in Jurkat cells (Supplementary Fig. 1). This indicated a direct interaction between miR-29b and DNMT3a/3b, and miR-142-5p, and DNMT1.

**Fig. 4:**
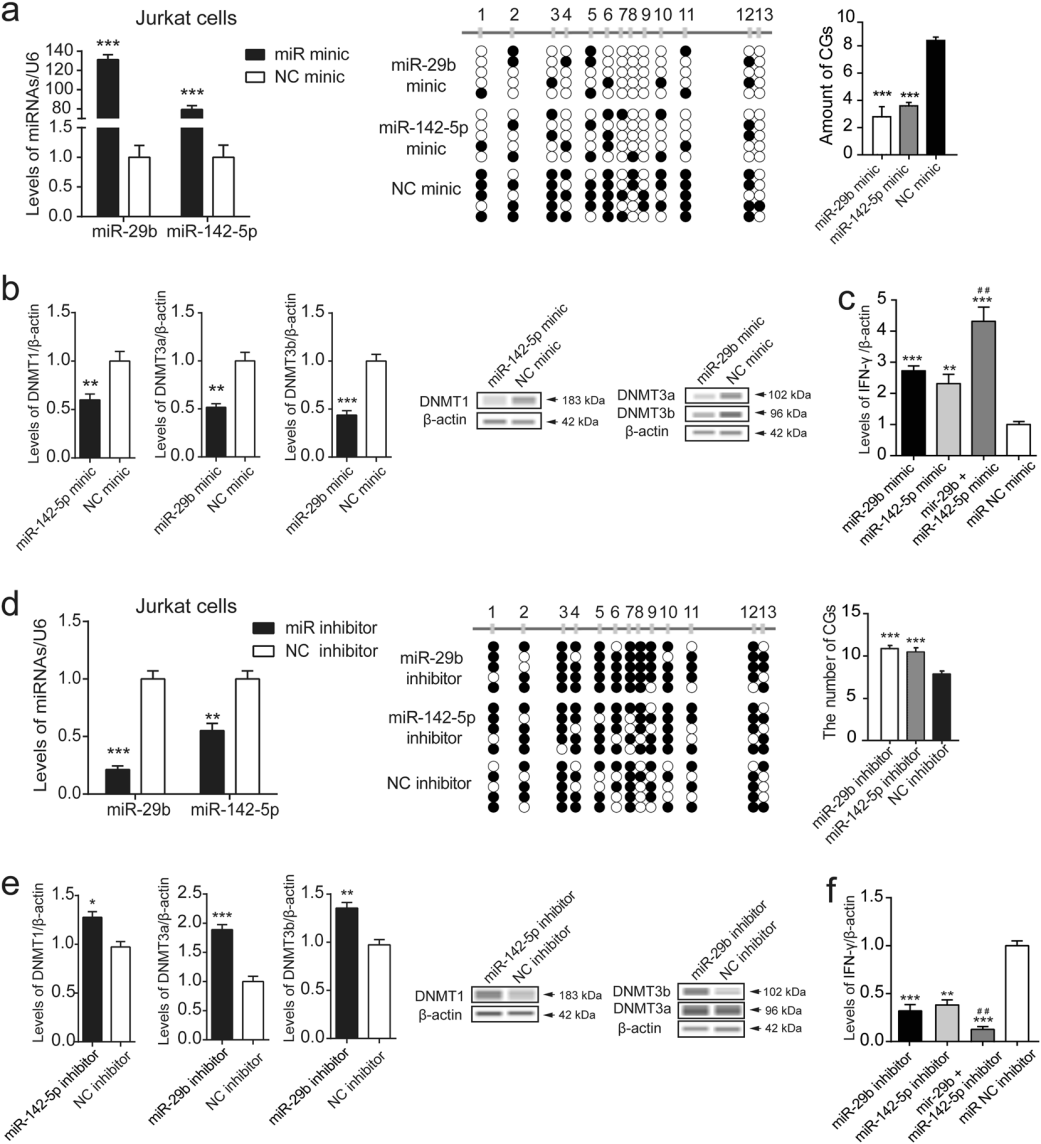
Overexpression of miR-29b/142-5p led to IFN-γ upregulation by targeting DNMTs. **a** Mimics of miR-29b/142-5p were transfected into Jurkat cells. Levels of miR-29b/142-5p were detected using qPCR and normalized to U6 levels. The methylation of the IFN-γ promoter were detected by bisulfite genomic sequencing in Jurkat cells. **b** Levels of DNMTs were detected by qPCR (normalized to β-actin) and western blotting. **c** Levels of IFN-γ were detected by qPCR and normalized to β-actin. **d** MiR-29b/142-5p inhibitors were transfected into Jurkat cells. Levels of miR-29b/142-5p were detected using qPCR and normalized to U6 levels. The methylation of the IFN-γ promoter were detected. **e** Levels of DNMTs were detected by qPCR (normalized to β-actin) and western blotting. **f** Levels of IFN-γ were detected by qPCR and normalized to β-actin. *p < 0.05, **p < 0.01, ***p < 0.001 vs. miRNA NC mimic or inhibitor. ^##^*p* < 0.001 vs. miRNAs mimic or inhibitor. Date represent mean values ± SD from three independent experiments

By transfecting mimics of miR-29b/142-5p into Jurkat cells, we evaluated the biologic role of miR-29b/142-5p. Overexpression of miR-29b/142-5p markedly suppressed expression of DNMT1, DNMT3a, and DNMT3b compared to the negative control. Furthermore, the methylation of LINE-1, ALU, and SAT2 sequences (Supplementary Fig. 2), and the *IFN*-γ promoter region were remarkably downregulated, which was associated with elevated IFN-γ expression (Fig. 4a, b, c). After transfecting the miRNA inhibitors, the levels of DNMTs were significantly elevated (*p* < 0.01), and the methylation of the *IFN*-γ gene promoter region was upregulated, while IFN-γ expression levels were markedly suppressed in Jurkat cell (Fig. 4d, e, f).

**Fig. 5:**
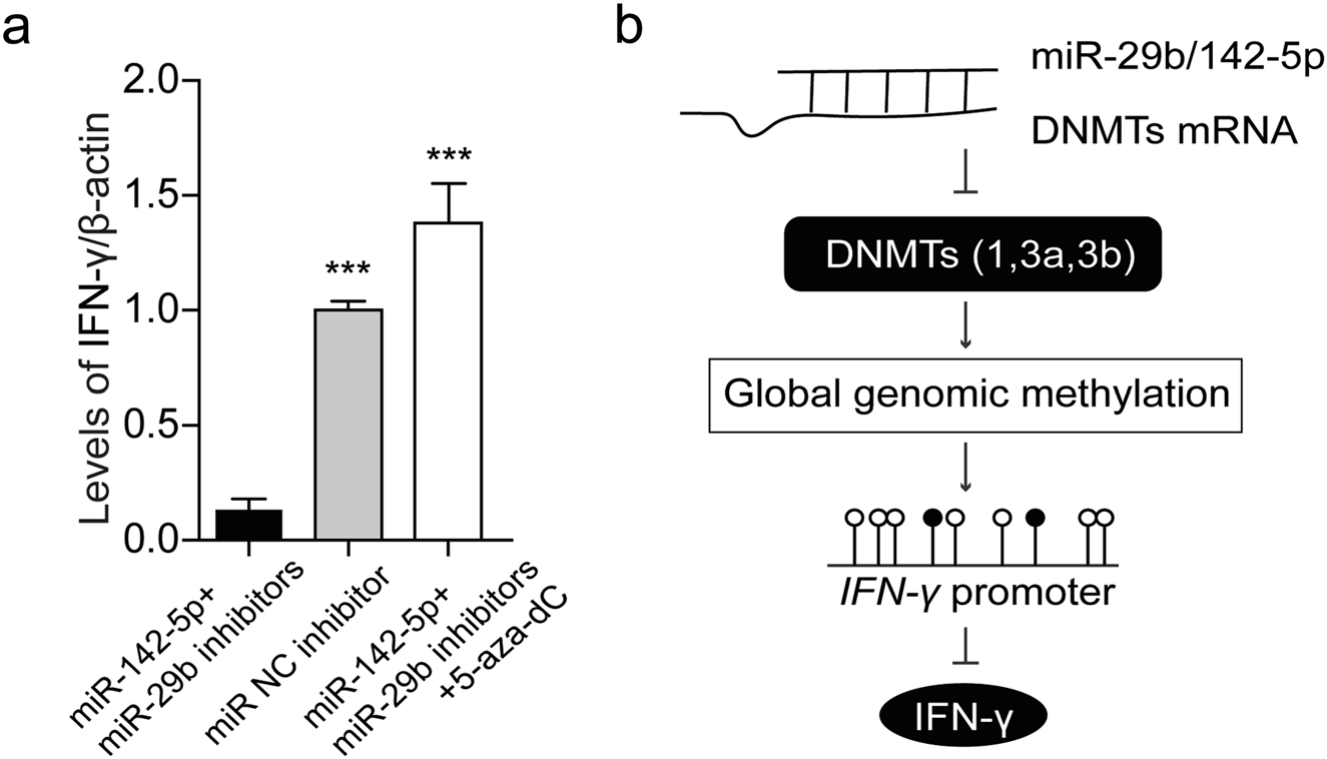
Effect of miR-29b/142-5p on IFN-γ expression. **a** The inhibitors of miR-29b/142-5p and 10 µM 5-aza-dC were transfected into Jurkat cells for 48 h. Levels of IFN-γ were detected by qPCR and normalized to β-actin. **b** Diagram summarizing the findings. ***p < 0.001 vs. miRNA co-inhibitors. Date represent mean values ± SD from three independent experiments

To further verify the effect of miR-29b/142-5p on IFN-γ expression, the two miRNA mimics or inhibitors were transfected into Jurkat cells at the same time. The levels of IFN-γ mRNA were elevated in miR-29b/142-5p co-mimics group than other three groups (Fig. 4c), while IFN-γ mRNA were decreased in miRNA co-inhibitors group than other three groups (Fig. 4f). In addition, the miR-29b/142-5p co-inhibitors and 5-aza-dC were transfected into Jurkat cells for 48 h. We found the expression of IFN-γ were higher in miR-29b/142-5p co-inhibitors with 5-aza-dC group than the miRNA co-inhibitors group (Fig. 5a). However, there was no significant difference compared with miRNA NC inhibitor group (Fig. 5a). These results indicate that miR-29b/142-5p could regulate the expression of IFN-γ by targeting DNMTs (Fig. 5b).

## Discussion

Numerous studies have shown that miRNAs, which are mainly involved in cellular regulation at post-transcriptional levels, are associated with a variety of biological processes, which include fetal development, organ formation, and cell proliferation and apoptosis^14–16^. Friedman et al. found that miR-29 was involved in the regulation of biliary atresia in a mouse model through its targeting of the *Igf1* gene^17^. In 2013, Japanese scholars reported that miR-29a/b1 could downregulate the levels of DNA methylation by targeting DNMT3, and found that miR-29b was closely associated with type I collagen synthesis during cirrhosis^18, 19^. Sonkoly et al.^20^ found that miRNA-142-5p was significantly increased in some autoimmune diseases. In the present study, miRNA microarrays showed that miR-29b and miR-142-5p overexpression was present in the liver and peripheral blood samples of biliary atresia patients. Closely related to DNA methylation, luciferase assays confirmed that these miRNAs target DNMT genes (*DNMT1*^21^, *DNMT3a*, and *DNMT3b*^22^).

DNA methylation is currently one of the most studied and important forms of epigenetic modification^23, 24^. DNA methyltransferase can be inhibited or destroyed by a variety of causes leading to genome-wide hypomethylation status^25^. DNA hypomethylation exists in numerous autoimmune diseases, which can promote the overexpression of methylation-sensitive genes, such as: *PRF1*, *IFN-γ*, *CD70*^26^. It can also lead to over-activation of T cells caused by structural changes to the chromosomes associated with T cells^27^. Activated CD4+ Th1 cells secrete large amounts of IFN-γ and induce a strong immune response. Biliary atresia is also commonly recognized as an autoimmune disease, which induces the secretion of large quantities of IFN-γ. This eventually leads to intrahepatic bile duct injury^6^. Our study showed that DNMT1, DNMT3a, and DNMT3b expression is significantly decreased, while the expression level of IFN-γ is increased in liver samples of biliary atresia cases.

A large number of repetitive sequences account for about 45% of the entire sequence of the human genome. Of these, the ALU sequence accounts for about 10% of the entire genome and consists of 1 million copies. The LINE-1 sequence is also a very important repeat sequence in the genome. The methylation status of these repeats is closely related to the overall methylation status of the genome and can, therefore, be used to measure the global DNA methylation levels^28^. By measuring the methylation status of the ALU, LINE-1, and SAT2 repetitive sequences^29^, we found that the methylation levels were significantly lower in biliary atresia cases than those in controls group. It could consequently be stated that DNA methylation levels were reduced globally in samples from biliary atresia patients. In addition, we found that the methylation levels of LINE-1 did not change, but that ALU and SAT2 were significantly altered after transfection with DNMT3a siRNA, mimics of miR-29b, and an inhibitor of miR-142-5p. According to the research by Daniel et al.^28^, the correlation coefficient between global methylation level and ALU combined with SAT2 is 0.85, *p* < 0.0001, which is to say that an elevation or decrease in both ALU and SAT2 may indicate a corresponding increase or decrease in global methylation levels.

Using 5-aza-dC to inhibit genomic methylation of Jurkat and LO2 cells^30^, we found that IFN-γ secreted from Jurkat cells increased by at nearly 350-fold, and confirmed that IFN-γ was mainly produced by T lymphocytes^31^. Therefore, Jurkat cells, which resemble human leukemia T cells, were transfected with DNMTs siRNA. The results showed that the global methylation of genomic DNA was reduced, and the methylation of IFN-γ promoter was decreased. This led to the overexpression of IFN-γ. To further investigate the effect of miR-29b/142-5p overexpression on IFN-γ, Jurkat cells were transfected with a mimic of miR-29b/142-5p. The expression of DNMT mRNA and proteins was decreased, while the methylation level of the *IFN*-γ promoter was decreased. This resulted in the abundant expression of IFN-γ. This was consistent with the hypothesis that biliary atresia pathogenesis is associated with increased miR-29b/142-5p expression and T-lymphocyte activation. This is further hypothesized to lead to elevated concentrations of IFN-γ, which induces or aggravates biliary atresia. Similarly, we found that miRNA inhibitors significantly increased DNMTs expression in Jurkat cells, which resulted in decreased expression of IFN-γ.

In summary, this study found that miRNA-29b/142-5p overexpression and targeted inhibition of DNMTs expression resulted in decreased overall gene methylation and overexpression of the methylation-sensitive *IFN*-γ gene. This may induce or aggravate the occurrence of biliary atresia. This study provides some evidence that further enhances our understanding of biliary atresia from the perspective of epigenetics while simultaneously exploring the pathogenesis of biliary atresia.

## Materials and methods

### Patient samples and cells

Sixty patients with type III biliary atresia and 20 patients with choledochal cysts who were treated at Children’s Hospital of Fudan University from October 2013 to July 2014 were enrolled (Table 1). All patients were diagnosed with biliary atresia by exploratory laparotomy with operative cholangiography, and underwent successful Kasai portoenterostomy. Choledochal cysts patients without jaundice have normal liver function served as controls. This study was approved by the Human Ethics Boards at Children’s Hospital of Fudan University. Written informed consent was obtained from the legal guardians of all subjects before starting study procedures. For RNA and protein extraction, tissue and blood samples were immediately snap-frozen and stored at −80 °C.

**Table 1:** Characteristics of patients with biliary atresia (BA) or choledochal cysts (CCs)

	BA	CCs	*p*-value
Age (months)	2.1 ± 0.6	18.5 ± 3.2	>0.05
Male	35	11	N/A
Female	25	9	N/A
Type	III^a^	I^b^	N/A
ALP (IU/L)	625.5 ± 28.7	77.6 ± 12.4	<0.05
ALT (IU/L)	122.6 ± 20.2	12.6 ± 5.6	<0.05
AST (IU/L)	176.4 ± 22.4	18.3 ± 7.2	<0.05
DBIL (umol/L)	121.1 ± 6.2	3.2 ± 1.2	<0.05
TBIL (umol/L)	145.1 ± 12.2	7.8 ± 2.3	<0.05
GGT (IU/L)	686.8 ± 86.4	36.4 ± 13.6	<0.05
TBA (umol/L)	126.2 ± 8.2	7.5 ± 2.2	<0.05

^a^Type III atresia refers to the discontinuity of both right and left hepatic ducts to the level of the porta hepatis

^b^Type I is the most common (80–90%) involving saccular or fusiform dilatation of a portion or entire common bile duct with normal intrahepatic duct. CCs patients without jaudice have normal liver function as controls

The Jurkat human leukemia T-cell line and LO2 human normal liver cell line were obtained from the Institute of Biochemistry and Cell Biology at the Chinese Academy of Science (TCHu 69, Shanghai, China). All the cells were cultured in RPMI-1640 supplemented with 10% FBS, 100 IU/mL penicillin, and 100 mg/mL streptomycin sulfates. Treated daily with 5-aza-dC (Sigma-Aldrich, Steinheim, Germany) at a final concentration of 10 μM for 24, 48, and 78 h, Jurkat and LO2 cells were seeded at a density of 1 × 106 cells/mL and 5 × 105 cells/mL, respectively.

### miRNA expression profiling

Total miRNAs were extracted from tissue and blood samples according to the manufacturer’s instructions by using mirVana miRNA isolation kit (Applied Biosystems, Foster City, CA, USA). As described in our previous study^32^, expression of miRNA was determined using miRNA microarrays (Agilent Technologies Inc., Santa Clara, CA, USA). Then, differentially expressed miRNAs were identified using the paired *t*-test with the cutoff criteria of *p* < 0.05 and |fold change| > 1.5. In order to ensure the screened differentially expressed miRNAs were accurately identified, hierarchical clustering analysis of samples was employed using heatmap.2 function of the gplots package in R^25^ based on the expression values.

### RNA extraction and quantitative reverse transcription PCR

Total RNA was extracted from cells or tissue with TRIzol reagent (Invitrogen, Carlsbad, CA, USA). Total miRNA quantification was achieved by quantitative reverse transcription PCR (qRT-PCR) using the miScript SYBR Green PCR Kit (Qiagen, Hilden, Germany) and the ABI 7500 Real-Time PCR System (Applied Biosystems). The U6 small nuclear RNA served as a reference. DNMT1, DNMT3a, DNMT3b, and IFN-γ mRNA levels were determined using SYBR Green method, with β-actin as an internal reference. Primer sequences are shown in Supplementary Table 1. The qRT-PCR results were analyzed with the 2^–ΔΔCt^ method.

### Western blot analysis

Snap-frozen liver samples and cells were homogenized in RIPA lysis buffer (Thermo Fisher Scientific, Hudson, NH, USA). Equal amounts of protein were subjected to sodium dodecyl sulfate polyacrylamide gel electrophoresis followed with wet transfer to nitrocellulose membranes. The membranes were blocked with 5% skim milk, and incubated with the primary antibodies for the DNMT1, DNMT3a, DNMT3b, IFN-γ and β-actin proteins (Abcam, Cambridge, MA, USA). After being incubated with a corresponding horseradish peroxidase-coupled secondary antibody (Beyotime, Shanghai, China), the membrane was allowed to react with the Lumi-Light ECL substrate (Thermo Fisher Scientific). ImageJ was then used to analyze the western blot result.

### DNA extraction and methylation analysis

Genomic DNA was isolated from liver tissues, Jurkat and LO2 cells using the DNA Isolation kit (Tiangen, Beijing, China) according to the manufacturer’s protocol. Determination of bisulfite conversion was performed using the EpiTect Bisulfite Kit (Qiagen). LINE-1, ALU-M2, and SAT2-M1 methylation levels were measured through Methylation-specific PCR analysis as described in previous study^33^. Primer sequences for each repetitive sequence is as follows: LINE-1-M1 (forward: 5′-GGACGTATTTGGAAAATCGGG-3′; reverse: 5′-AATCTCGCGATACGCCGTT-3′), ALU-M2 (forward: 5′-GCGCGGTGGTTTACGTTT-3′; reverse: 5′-AACCGAACTAATCTCGAACTCCTAAC-3′), and SAT2-M1 (forward: 5′-TCGAATGGAATTAATATTTAACGGAAAA-3′; reverse: 5′-CCATTCGAATCCATTCGATAATTCT-3′). The bisulfite sequencing PCR reaction system was composed of 10 × PCR buffer, 5.25 μM dNTP Mix, 0.5 μM of each primer, 0.75 U hot start DNA polymerase, and 20 ng bisulfite modified DNA. Products from bisulfate PCR reactions were analyzed on 2% agarose gels and purified using QIAquick Gel Extraction Kit (Qiagen). Each purified product was cloned into a pMD 19-T Vector (Takara, Tokyo, Japan) and then transfected into *Escherichia coli* DH5α competent cells (Vazyme Biotech Co., Piscataway, NJ, USA). Five clones from each sample were sequenced (Shinegene Molecular Biotechnology Co., Shanghai, China).

### Luciferase reporter assay

DNMT (DNMT1, DNMT3a, and DNMT3b) mRNA 3ʹ-UTR fragments containing the putative miR-29b/142-5p-binding sequence were amplified through PCR and cloned downstream of the luciferase reporter gene between the *Xba*I and *Eco*RI cutting sites of the pGL3-control vector. The primers used for the DNMT-3ʹ-UTR clones are listed in Supplementary Table 2. Jurkat cells were co-transfected with pGL3-DNMT-3ʹ-UTR or pGL3-DNMT-3ʹ-UTR-mut, with cell extracts prepared 24 h after transfection. Luciferase activity was measured with the Dual-Luciferase Reporter Assay system (Promega, Madison, WI, USA) according to the manufacturers’ protocol.

### miRNA target prediction

At least two databases of the following five usual prediction databases: TargetScan (http://www.targetscan.org) and MiRanda (http://www.microrna.org/microrna/home.do), PicTar (http://pictar.mdc-berlin.de/), MirTarget2 from miRDB (http://mirdb.org/miRDB/ download.html) and PITA (http://genie.weizmann.ac.il/pubs/mir07/mir07_prediction.html) were used to predict miRNA targets and conserved sites bound by the seed region of miR-29b and miR-142-5p in silico.

### Transfection

Jurkat cells were transfected with either 20 nmol/L DNMT siRNAs or negative control (NC) siRNA (Biotend, Shanghai, China), 20 nmol/L of one of the following: a mimic of miR-29b/142-5p, or an inhibitor of miR-29b/142-5p, or a mimic/inhibitor NC, and CY3 dye as positive control for 48–72 h. These were achieved using Lipofectamine 2000 Transfection Reagent (Thermo Fisher Scientific) according to the manufacturer’s protocol. The transfection efficiency of DNMT siRNAs and miRNA mimic or inhibitor were >90%.

### Statistical analysis

Data were presented as the means ± standard deviation of at least three experiments. Statistical analysis was performed using SPSS 19.0 (SPSS, Chicago, IL, USA). An unpaired *t*-test was applied for statistical comparison between biliary atresia and choledochal cysts groups, DNMT siRNAs and negative control groups, and miRNA mimic or inhibitor and negative control groups. Analysis of variance was used to analyze different time points of 5-aza-dC groups. Pearson’s correlation coefficient was used for correlation analysis between miRNAs and DNMTs expression. *p*-value < 0.05 was considered statistically significant.

## Electronic supplementary material

Supplementary Figure 1

Supplementary Figure 2

Supplementary Table 1

Supplementary Table 2

Supplementary Figure legends
