# Supplementary figures and images for: MicroRNA-29b/142-5p contribute to the pathogenesis of biliary atresia by regulating the IFN-γ gene
Source: Cell Death Dis. 2018 May 10;9(5):545. doi: 10.1038/s41419-018-0605-y (PMC5945737; doi:10.1038/s41419-018-0605-y)

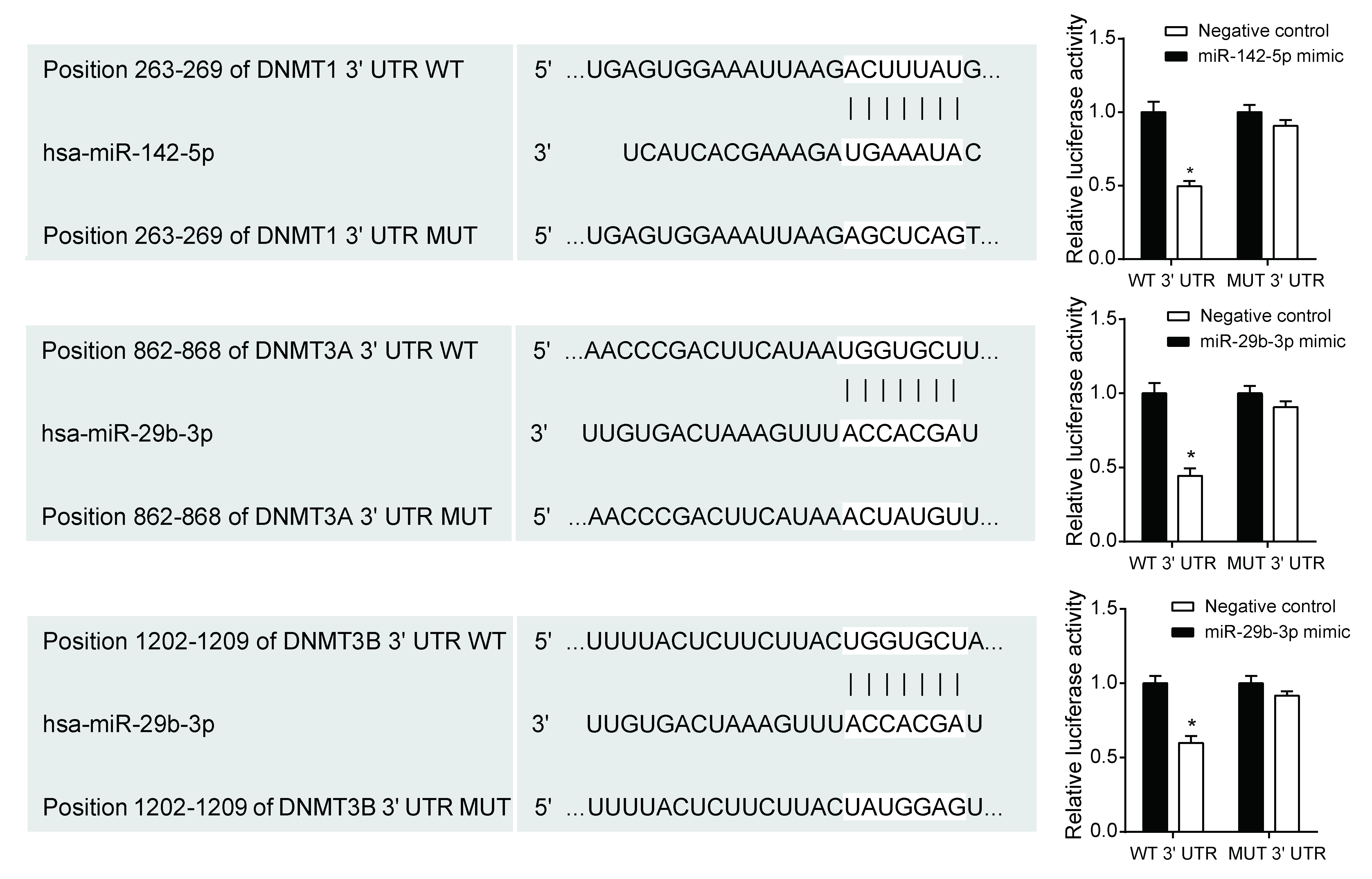

Supplement: Supplementary file 1 — Supplementary Figure 1 [file 41419_2018_605_MOESM1_ESM.tif]

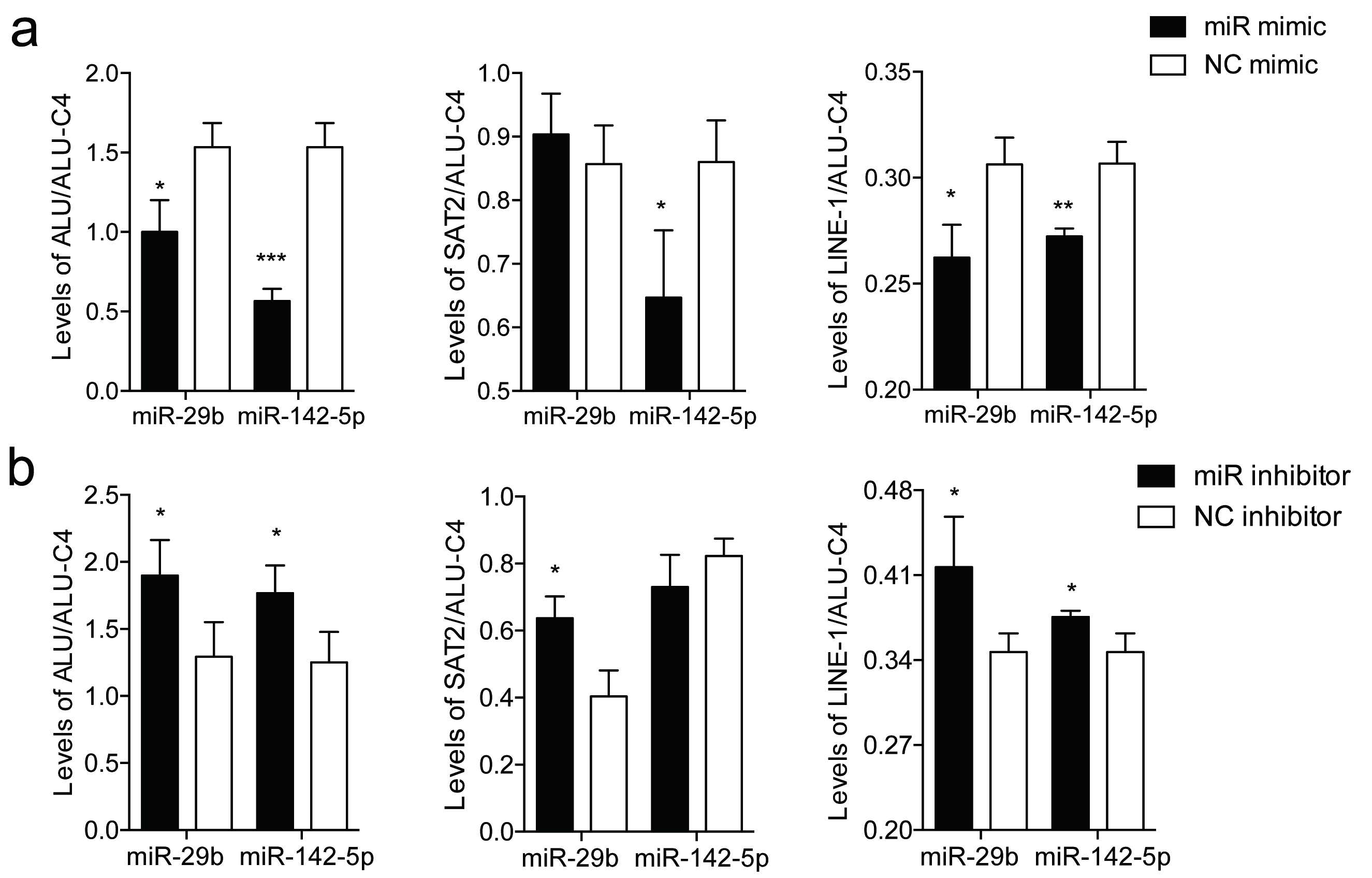

Supplement: Supplementary file 2 — Supplementary Figure 2 [file 41419_2018_605_MOESM2_ESM.tif]
