# Supplementary material for: MicroRNA-29b/142-5p contribute to the pathogenesis of biliary atresia by regulating the IFN-γ gene
Source: Cell Death Dis. 2018 May 10;9(5):545. doi: 10.1038/s41419-018-0605-y (PMC5945737; doi:10.1038/s41419-018-0605-y)
Supplement: Supplementary file 3 — Supplementary Table 1 [file 41419_2018_605_MOESM3_ESM.docx]

Supplementary Table 1 Primer sequences for qRT-PCR

| Gene |  | Sequences（5’-3’） |
| --- | --- | --- |
| IFN-γ | Forward | 5'-TGGCTTTTCAGCTCTGCATCGT-3'(22 bp) |
| IFN-γ | Reverse | 5'-TCCACACTCTTTTGGATGCTCTGGT-3'(25 bp) |
| DNMT1 | Forward | 5'-GTGGGGGACTGTGTCTCTGT-3'(20 bp) |
| DNMT1 | Reverse | 5'-TGAAAGCTGCATGTCCTCAC-3'(20 bp) |
| DNMT3a | Forward | 5'-CAGCTTCCACGTTGCCTTCT-3'(20 bp) |
| DNMT3a | Reverse | 5'-CATCTGCAAGCTGTCTCCCTTT-3'(22 bp) |
| DNMT3b | Forward | 5'-TACACAGACGTGTCCAACATGGGC-3'(24 bp) |
| DNMT3b | Reverse | 5'-GGATGCCTTCAGGAATCACACCTC-3'(24 bp) |
| β-actin | Forward | 5'-TCCTCCCTGGAGAAGAGCTA-3'(20 bp) |
| β-actin | Reverse | 5'-GTACTTGCGCTCAGGAGGAG-3'(20 bp) |
