# Supplementary material for: MicroRNA-29b/142-5p contribute to the pathogenesis of biliary atresia by regulating the IFN-γ gene
Source: Cell Death Dis. 2018 May 10;9(5):545. doi: 10.1038/s41419-018-0605-y (PMC5945737; doi:10.1038/s41419-018-0605-y)
Supplement: Supplementary file 4 — Supplementary Table 2 [file 41419_2018_605_MOESM4_ESM.docx]

Supplementary Table 2 Primer sequences for the DNMTs-3ʹ-UTR clones in luciferase reporter assay

| Gene | Sequences (5’-3’) | Length of products |
| --- | --- | --- |
| DNMT1-WT-F | TAG GAATTC TTGGCTGACATGAAGC | 215bp |
| DNMT1-WT-R | TAG TCTAGA AGGAGAGATTTATTTGAAG |  |
| DNMT1-MUT-R | ACTAC AGCATCA CTTAATTTCCACTC | 167bp |
| DNMT1-MUT-F | GAGTGGAAATTAAG TGATGCT GTAGT | 74bp |
| DNMT3a-WT-F | TAG GAATTC AACCCAGTTAGCAGCAG | 362bp |
| DNMT3a- WT-R | TAG TCTAGA GCTCCCAAGTTCTCCTC |  |
| DNMT3a-MUT-R | CGCTGTTTGAA ACTATGT TTATG | 221bp |
| DNMT3a-MUT-F | CATAA ACATAGT TTCAAACAGCG | 182bp |
| DNMT3b-WT-F | TAG GAATTC GACAGCAGTCAGGGACAG | 408bp |
| DNMT3b-WT-R | TAG TCTAGA TCCGTCATCTTTCAGCC |  |
| DNMT3b-MUT-R | TCTACAAAA TATGGAGT GTAAGAAG | 237bp |
| DNMT3b-MUT-F | CTTCTTAC ACTCCATA TTTTGTAGA | 224bp |

F, forward primer; R, reverse primer.
