# Supplementary material for: MicroRNA-29b/142-5p contribute to the pathogenesis of biliary atresia by regulating the IFN-γ gene
Source: Cell Death Dis. 2018 May 10;9(5):545. doi: 10.1038/s41419-018-0605-y (PMC5945737; doi:10.1038/s41419-018-0605-y)
Supplement: Supplementary file 5 — Supplementary Figure legends [file 41419_2018_605_MOESM5_ESM.docx]

**Supplementary Figure legends**

**Supplementary Figure 1.** Luciferase reporter assays of miR-29b/142-5p. Transfection with mimics of miR-29b and miR-142-5p could respectively inhibit the DNMTs wild type vectors luciferase activity (p<0.05), but failed to inhibit DNMTs mutant vectors luciferase activity in Jurkat cells. *p < 0.05. Date represent mean values ± SD from three independent experiments.

**Supplementary Figure 2.** The quantitative methylation of the LINE-1, ALU and SAT2 sequences were analyzed by Methylation-specific PCR and normalized to ALU-C4 in Jurkat cells after transfected with miRNA mimic (a) and inhibitor (b). *p < 0.05, **p < 0.01, ***p<0.001. Date represent mean values ± SD from three independent experiments.
